# Primary Gastrointestinal Involvement in a Case of Extranodal-Extranasal Natural Killer T Cell Lymphoma

**Published:** 2020-01

**Authors:** Somayeh Lookzadeh, Mihan Pourabdollah Toutkaboni, Hamidreza Jamaati, Mitra Rezaei, Mehran Marashian

**Affiliations:** 1 Chronic Respiratory Diseases Research Center, National Research Institute of Tuberculosis and Lung Diseases (NRITLD), Shahid Beheshti University of Medical Sciences, Tehran, Iran,; 2 Virology Research Center, NRITLD, Shahid Beheshti University of Medical Sciences,Tehran, Iran.

**Keywords:** Natural killer cell, T cell, Lymphoma, Extranasal, Extranodal

## Abstract

Extra-nasal types of Extra-nodal natural killer cell lymphoma (ENKL) have been known with poorer prognoses than nasal type with the worst responses to treatment. The current work introduces a case of ENKL with GI involvement with no nasal manifestations.

We report a 56-year male farmer with fever, productive cough, dyspnea, anorexia, vomiting and chill in addition to malaise and cachexia of three months duration referred to a hospital with acute abdominal pain, and was diagnosed as peritonitis due to perforated terminal ileum ulcer before experiencing surgery as a case of acute abdomen. The pathologic study of the relevant biopsy showed “ulceration and necrosis with dense fibrinoleukocytic exudation and granulation tissue formation. CT scan determined a bilateral mass like haziness which was more likely to be metastatic. The review of the previous pathologic specimens raised Natural Killer/T cell Lymphoma (NKTL), the reason for which we focused on the patient’s sinuses and nasal area as well as nasopharynx. There was no finding in examination and endoscopy of sinuses. Pathology also found malignant high grade non-Hodgkin T cell lymphoma in specimens obtained from debridement of ulcer at terminal ileum. It also showed that most of the tumor cells were positive for CD3, CD56, CD8, and LCA but negative for CD19, CD20 and AE1/AE3. Positive reactions for CD30 were shown by some cells. CD56, CD3, and CD8 were expressed by neoplastic cells and CD30 were positive in few cells. Proliferative activity (Ki67 index) was high (60–70%). This was the main base to diagnose an extra-nodal extra-nasal NK/T cell lymphoma.

In conclusion, Intestinal changes at middle age, especially in men with nonspecific clinical manifestations is highly advised to be studied pathologically and genetically for T cell types like CD30 positive T cells which are usually engaged in ENKTL.

## INTRODUCTION

Lymphomas, as clonal neoplasms involving B cells, T cells and natural killer cells in different stages of maturation, contribute to 4% of malignancies in Western countries and the fifth most common cancer resulting in death (1,2). The World Health Organization (WHO) has classified NK-cell tumors into three main types including extra-nodal NK/T-cell lymphomas (containing nasal and extra-nasal), NK-cell leukemia, and finally blastic variants of hematodermic neoplasms (3). Extra-nodal NK/T cell lymphoma (ENKTL) is a localized type of the mentioned conditions which mostly involves midline facial structures like nasal cavity as well as nasopharynx. Extra-nodal natural killer cell lymphoma (ENKL) is more common at median age of 50 years, more frequent in males and is more prevalent in Asia, Central and South America as Suzuki et al. realized in 2005 (4). Studies show 80 upper aerodigestive tract (UAT) involvements mainly including nasal cavity, nasopharynx, oral cavity, oropharynx and hypopharynx (5). However, 10% of ENKL cases are usually extra-nasal with a predilection for involvement of GI tract, skin, salivary glands, liver, spleen, adrenals, and testis (6–10).

Extra-nasal types of ENKTL have been known with poorer prognoses than nasal type (7) among which, studies showed the worst responses to treatment in cases with skin and soft tissue engagement (11). Gastrointestinal involvement is usually known as a rare type of ENKTL and there is no established therapeutic strategy for GI tract involving ENKTL, yet.

Pathologic wise, NK-cell tumor is classically a polymorphic infiltration along with angioinvasion and cytoplasmic azurophilic granules. Necrosis, apoptosis, and background inflammation are usually seen at the site of involvement which contains plasma cells, small lymphocytes, histiocytes, and eosinophils. (3) ENKTL immunophenotype is clearly similar to that of NK cell. Most of the time, CD2, CD56 and cytoplasmic CD3 are expressed by atypical cells while in uncommon cases, CD4, CD8 and CD7 expression may occur (12, 13).

In terms of diagnostic approaches, like other lymphomas, unique anatomic sites of involvement could raise dedicated imaging studies, especially in nasal cavity, hard palate, and anterior fossa as Kohrt et al. figured out before (3). Furthermore, PET scan presents greater than 95% sensitivity except in cutaneous or bone marrow involvement (14, 15).

The current work introduces a case of extra-nodal natural killer cell lymphoma with GI involvement with no nasal manifestations.

## CASE SUMMARIES

The patient was a 56-year male farmer with fever, productive cough, dyspnea, anorexia, vomiting and chill in addition to malaise and cachexia of three months duration, who referred to a hospital with acute abdominal pain, to be diagnosed as peritonitis due to perforated terminal ileum ulcer before experiencing surgery as a case of acute abdomen. The pathologic study of the relevant biopsy showed “ulceration and necrosis with dense fibrinoleukocytic exudation and granulation tissue formation”. He started complaining of solid dysphagia along with respiratory complaints after antibiotic therapy for pneumonia two months before referring to hospital and finally got better. In the mentioned center, he was endoscopically evaluated and pathology reported “esophageal mucosa with ulceration, severe acute and chronic inflammation”. He was smoker (40 pack/year) but no relevant medical history or family history was found. Vital signs were not very stable: HR=96/min; T=37.8 °C; RR=24/min and O_2_ sat=81%. Physical examination showed bilateral rales, 2+ edema in right lower extremity, dyspnea and right side Deep vein thrombosis (DVT) was confirmed through doppler ultrasound.

CT scan determined bilateral mass like haziness which was more likely to be metastatic. Mediastinal lymphadenopathy (LAP) and mild pericardial effusion were seen and despite the patient’s general situation which put CT-guided lung biopsy and bronchoscopy at moderate to high risk; CT-guided biopsy was done to show only necrosis at the studied site. Due to lack of evidence for confirmed diagnosis, the previous pathologic specimens were demanded from the previous center of hospitalization in order to double check. The review of the previous pathologic specimens raised Natural Killer/T cell Lymphoma (NKTL). This was why we focused on the patient’s sinuses and nasal area as well as nasopharynx. There was no finding in examination and endoscopy of sinuses. Pleural fluid was studied pathologically and there was no finding compatible to malignancy. There was no finding through **Bone marrow** aspiration (BMA) and **Bone marrow** biopsy (BMB) for bone marrow involvement. This was while CT guided lung biopsy raised high grade CD30 positive T cell lymphoma. Pathology also found malignant high grade non-Hodgkin T cell lymphoma in specimens obtained from debridement of ulcer at terminal ileum. This was the main base to diagnose an extra-nodal NK/T cell lymphoma for the patient and chemotherapy was started on the 20^th^ day of the current hospitalization in our center.

**Figure 1.**
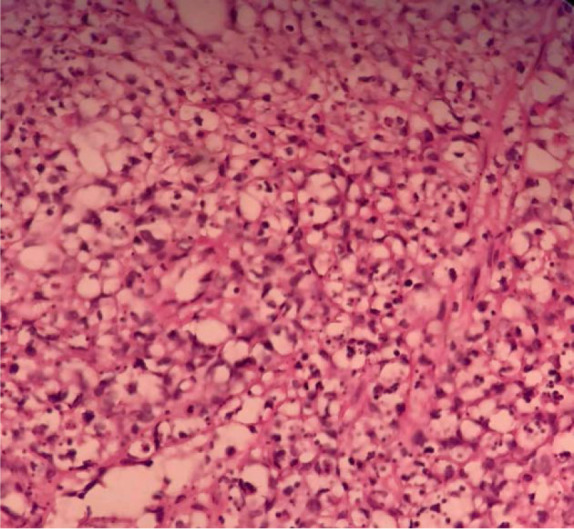
Ileal wall, infiltration of pleomorphic neoplastic cells with light eosinophilic to clear cytoplasm (X400).

Specimen obtained from terminal ileum showed necrosis and ulceration as well as heavy transmural infiltration of pleomorphic neoplastic lymphoid cells with light eosinophilic to clear cytoplasm, some with prominent nucleoli including few multinucleated ones with bizarre nuclei (Figure 1). Most of the tumor cells were positive for CD3, CD56, CD8, and LCA but negative for CD19, CD20 and AE1/AE3. Positive reactions for CD30 were shown by some cells.

Esophageal specimen showed fragments of squamous mucosa with severe chronic inflammation, comprised of small bland looking lymphocytes and some infiltrative adhesive atypical cells. Areas of necrosis were also identified in esophageal mucosa. CD56, CD3, and CD8 were expressed by neoplastic cells and CD30 was positive in few cells. Proliferative activity (Ki67 index) was high (60–70%).

**Figure 2.**
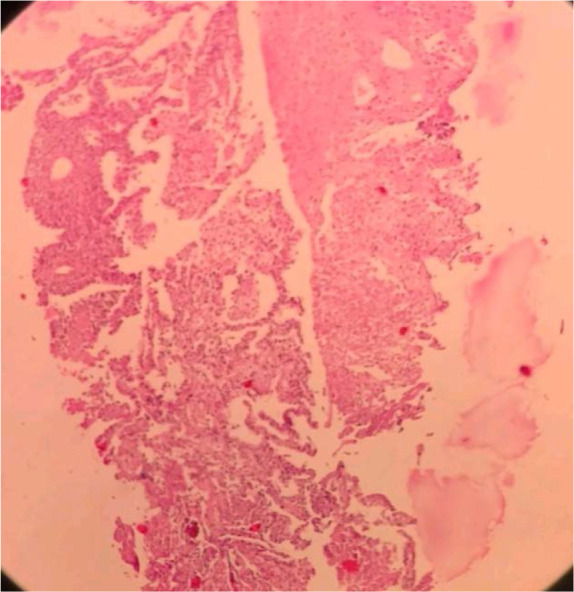
Lung parenchyma with extensive necrosis infiltrated by pleomorphic neoplastic cells (X100).

**Figure 3.**
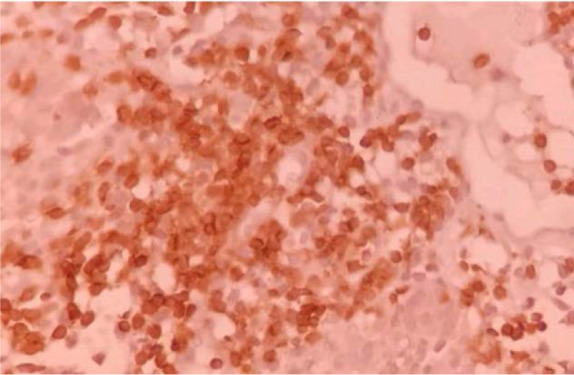
Predominantly cytoplasmic positive reaction of neoplastic cells for CD3 a pan T cell marker (X200).

**Figure 4.**
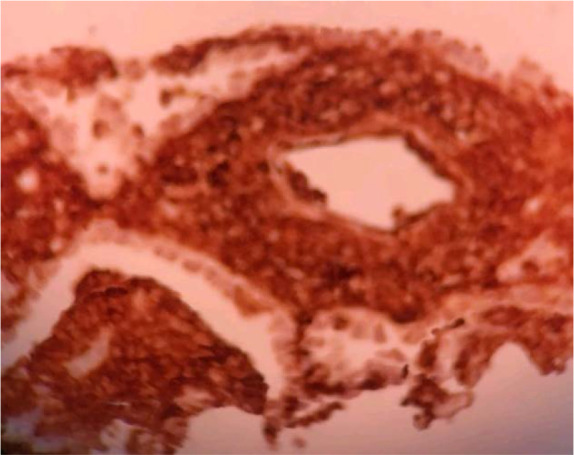
Lung biopsy, diffuse immunoreactivity of neoplastic cells for confirming presence of NK cells (X100).

CT-guided lung biopsy presented extensive areas of necrosis and patchy uneven interstitial thickening due to polymorphous cellular infiltration comprised of atypical adhesive cells with clear cytoplasm and pleomorphic atypical nuclei admixed with histiocytes including epithelioid ones, lymphocytes, few eosinophils and nuclear debris (Figure 2). Immunohistochemistry (IHC) staining demonstrated neoplastic cells positive for LCA, CD3, CD56, CD8, CD30 and EMA but negative for CK7, CK20, TTFI, AE1/AE3, CD19, CD20, CD5; while Ki67 proliferation index was high (70%) (Figure 3,4).

## DISCUSSION

The current case report presents a patient with GI tract involvement with non-Hodgkin lymphoma later diagnosed as extra-nodal/extra-nasal natural killer cell lymphoma with metastasis to lungs. In 2017, an article was published by Au et al. to compare nasal and extra-nasal natural killer/T cell lymphoma working on 136 cases through an international project. They found 68% nasal and 26% extra-nasal rates of cases. Among 35 primary extra-nasal cases of the mentioned study, the most sites of involvement were intestine (37%), while lung was also engaged in 14% which could justify the way our patient presented the manifestations (16). Elevated serum LDH was seen in our patient which was also seen in cases of Au et al. Our patient presented low blood cell count after few days of hospitalization which was similar to findings by Au et al. though neither they nor we identified any confirmed bone marrow involvement.

In 2013, Kim et al. published an article to discuss GI tract involvement in extra-nodal NKTL retrospectively in twelve centers in Asia from 1991 through 2012 (7). Among 81 patients with ENKTL, they reported only 22% nasal involvement and 78% had no nasal problem. Interestingly only 68% of the patients presented GI manifestations and male predominance was clearly seen, unlike some studies that do not generally consider a role for gender in this regard. Eighteen cases of nasal ENKTL in the named study were clearly diagnosed through nasal biopsy, while their GI tract involvement was evaluated via PET/CT scan studies. Like our case in addition to the study conducted by Au et al. (16), small intestine was the most damaged part of GI tract (70%) and 42% of their patients had only small intestine involvement. This study concluded similar findings to our report that ileum was the most common part of GI tract involved in ENKTL (29%). Our patient also had a mass in ileum, diagnosed as non-Hodgkin lymphoma.

In terms of prognosis, almost all the studies raise a poor prognosis for extra-nasal types. (16–19). For nasal ENKTL, clinical features and laboratory findings such as serum LDH level, hemoglobin, platelet count and C-reactive protein level have been shown to be predictive for the prognosis, although no clinical and histologic features play role in this regard for extra-nasal cases (20–23). However, due to difficult discrimination between intestinal involvement of ENKTL and inflammatory or infections, it is inevitable to have usually a delay in diagnosis, especially in extra-nodal/extra-nasal natural killer T cell lymphoma. That was also what happened for our case which caused diagnostic delay for more than three months. Diagnostic delay is chiefly due to non-specific clinical and endoscopic features. Focusing on histopathological and immunohistochemical findings as well as genetic studies particularly at advanced or middle ages could perfectly help achieve on time effective diagnosis.

Concerning C30 expressed T cells through lung biopsy, some previous studies have shown ENKTL types with CD30 expression, especially with spleen, prostate, adrenal glands and nasal involvement. CD56 is formally a nerve cell adhesion factor and could identify poor prognosis when expressed. CD30 is chiefly known as main player in pathology of ENKTL which is a perfect target for the treatment (12).

In the case introduced through the current report, GI tract pathology showed CD56 positive cells. Through their clinical study for CD56 positive immunophenotyped in ENKTL, YX et al. believed that nasal type, especially with upper GI tract involvement, expressed more CD56 than extra nasal one, although there was no difference in terms of clinical findings related to CD56 expression (24).

In conclusion, intestinal changes at middle age, especially in men with nonspecific clinical manifestations is highly advised to be studied pathologically and genetically for T cell types like CD30 positive T cells which are usually engaged in ENKTL.
